# “*Sometimes I just forget them*”: capturing experiences of women about free menstrual products in a U.S. based public university campus

**DOI:** 10.1186/s12905-023-02457-2

**Published:** 2023-07-04

**Authors:** Meghana Rawat, Allison Novorita, Jaclyn Frank, Stevie Burgett, Risa Cromer, Audrey Ruple, Andrea L. DeMaria

**Affiliations:** 1 Department of Communication, College of Humanities and Social Sciences, Utah Valley University, 800 W University Parkway, Orem, UT 84058 USA; 2 Department of Public Health, College of Health and Human Sciences, Purdue University, West Lafayette, IN USA; 3 Weldon School of Biomedical Engineering, College of Engineering, Purdue University, West Lafayette, IN USA; 4 Department of Anthropology, College of Liberal Arts, Purdue University, West Lafayette, IN USA; 5 Department of Population Health Sciences, Virginia-Maryland College of Veterinary Medicine, Virginia Tech, Blacksburg, VA USA

**Keywords:** Menstruation, Menarche, Period policy, Free tampons, Free pads, Period poverty

## Abstract

**Background:**

Studies have proven that lack of access to menstruation products negatively affects school attendance, academic performance, and individual health. Implementing “period policies,” or programs offering free menstruation products, are becoming popular in schools, businesses, and communities in high-income countries. U.S.-based Purdue University announced in February 2020 that free pads and tampons would be stocked in all women’s and gender-neutral restrooms in campus buildings. This study aimed to capture the experiences of menstruators about free menstrual products and the impact of a university-wide free menstruation management product policy and program. A second purpose was to understand how access to menstrual management products is intertwined with broader socio-cultural experiences of a menstruator.

**Methods:**

As part of a larger study, virtual focus group discussions (*n* = 32 across 5 focus groups) were conducted in February 2021. Eligible participants were student-menstruators attending Purdue University. We used thematic analysis techniques for data analysis, allowing for a constant comparative approach to data contextualization and theme identification.

**Results:**

Focus group discussions revealed vivid menarche and menstruation experiences, shifting period culture, recollections of shame and stigma, and use of various technologies to manage menstruation. Recommendations for community-based programs offering free products included maintaining stock, making informed product choices, and broadly distributing program information to increase awareness of free product placements.

**Conclusions:**

Findings offer practical recommendations that will contribute to menstruation management and period poverty solutions for university communities.

## Introduction

In recent years, United States (U.S) legislation is being called upon to do away with the “tampon tax,”: a luxury tax that is placed upon menstrual hygiene products [1]. Advocates supporting the luxury tax removal also advocate for providing menstrual hygiene products through welfare benefits [2]. In the fight for gender equality and human rights, this tax reform is considered by advocates as an “essential tool” [2]. Britain, Kenya, India, Canada, Australia and Germany are some countries that have eliminated the tax on menstrual hygiene products [3–5]. School leaders across the United States are urging US legislation to provide menstrual hygiene products for free to users of any women’s and gender-neutral restrooms in schools [6]. This provision of products should be accompanied by discussions surrounding education, mitigation of menstrual shame, and de-stigmatization [6, 7].

One argument maintains that public facilities, such as schools, should provide for menstrual needs as they would for urinary or defecatory hygiene requirements [8, 9]. Studies conducted in the US as recent as 2021 note that students miss school due to unaffordability of menstrual health products. This is specifically exacerbated in urban cities amongst BIPOC school aged girls in urban cities [10]. Campaigns for menstrual health and hygiene continue to gain momentum as they spark interest and activism [11]. For example, a qualitative study with school going girls in the Midwest highlighted that students find learning about menstruation, experiencing menstruation, and managing menstruation as interrelated issues when they start menstruating. These experiences are further made challenging due to social norms and how we understand the meaning of menstruation as culture [12].

### Study purpose

Menstruation goals in global health initiatives have been extended to include a global standardization of policies providing for menstrual hygiene management support, designation of responsibility to provide this support, and an increase in worldwide menstruation-related education [13]. On a national scale, United States policy and laws maintain the inaccessibility of menstrual hygiene products to some of the most vulnerable groups of menstruating individuals: those who experience poverty, unstable housing, and incarceration [14, 15]. Menstruation products are not provided in correctional facilities, are rarely included in funding for emergency/crisis centers and shelters, and are seldom exempt from sales tax in most US states [14, 16]. Activist organizations, student-led clubs, and state legislators alike have expressed their commitment to menstrual health equity.

In a study conducted with college students in the United States, Cardoso et al. [17] reported that among their small sample (*N* = 471), 14.2% of women had experienced period poverty in 2019 with 10% of participants reporting they experienced challenges in accessing affordable menstrual products every month. Their analysis also revealed that women who experienced accessibility stressors monthly reported moderate/severe depression, but they did not expand upon this finding. They called for universities and public facilities to provide free menstrual products to address the basic need of menstruators. As a result, “there are now more than 140 bills in 37 states, including 8 bills introduced on the federal level, to advance menstrual equity by requiring free access to period products, eliminating tax, and requiring ingredient disclosure.” (According to Women’s Voices for the Earth [18]). Moreover, recently six states have mandated having pads and tampons available in public schools, while 13 states have passed laws to require them in prisons, jails, and shelters [19].

However, providing free menstrual products comes with its own set of challenges. In their study, Gruer et al. [15] examined four universities across the United States each with a pilot program of free menstrual products. They found that universities need champions on their campus to launch such a policy and navigate funding issues to sustain the program. They specifically found that both student and administrative champions noted instances of students reporting their non-preparedness for periods and how that hampered them from attending classes. Similar results expounding the importance of policy champions and flexibility in distribution of free products were found in a case study in early implementation of free menstrual products in New York [20]. While many universities in the United States have followed the path of other high-income level (HIC) countries to provide free access to menstrual products in women’s and gender-neutral bathrooms, Gruer et al., [15] notes the need for more studies about other universities who have launched such programs.

### Purdue University policy

Purdue University’s Faculty Senate proposed free pads and tampons in all women’s and gender-neutral bathrooms on its February 2020 meeting agenda [21, 22]. Before a vote could take place, Purdue’s then President, Mitch Daniels, announced an executive decision to approve the initiative by providing free products in all women’s and gender-neutral bathrooms in campus buildings [23]. The Senate followed with a confirmatory vote less than two weeks later to affirm its support for the resolution and the Office of the Provost secured the funding needed [24]. Purdue began implementing this policy by retrofitting existing tampon and pad dispensers in restrooms to dispense products without charge. Modifying the machines was estimated at $30,000 [25]. Once individual machines were modified, they resulted in little financial impact compared to when they operated for profit [26]. All machines were modified and filled by the end of February 2020, shortly before the COVID-19 shutdown. In March 2021, the Faculty Senate voted to extend this policy and program to men’s restrooms in campus buildings [27].

### Goal of the study

This study was part of a larger study exploring free tampon and pad program and product access. Volunteers were recruited to sample various menstrual products that the university was willing to offer. By understanding their product preferences, volunteers gained insight into their reflection about the program, and its impact on their menstruation experiences.

The goal of this study is two-fold. First, it attempts to contribute to existing literature in high-income countries about the impact of accessible menstrual management products in public settings, especially expanding it to universities as majority still focus on middle and secondary public schools. Second, it attempts to highlight how experiences of menstruators are intertwined with the socio-cultural conversations around menstruations including accessibility of products in a university setting.

### Free menstrual management product policies and high-income countries

Many high-income countries such as Scotland have sought to fight the stigma surrounding menstruation by advocating to end period poverty by implementing a policy making free pads and tampons available to menstruators of all ages [28]. In the United States, a similar program in Brookline, Massachusetts started providing menstrual products at no cost to users in all town-owned restrooms beginning in July 2021 [29]. In education settings, schools in New Hampshire, Washington, and the city of Boston follow New York and Illinois, where laws already exist requiring grades six through twelve schools to provide free menstrual products in school restrooms [30, 31]. Menstruation management product pantries have been established at the university level to close gaps regarding access to appropriate menstrual hygiene products [32].

However, Schmitt et al. [10] reported gaps in BIPOC girls’ (age 15–19 years) practical knowledge and their lack of support around menstruation-related issues in urban cities such as New York City, Chicago, and Los Angeles. Participants also reported challenges with menstrual pain during school, and the negative impact of menstruation on their engagement in physical or sports-related activities. Similar issues with stigma were reported with girls between the ages of 12–16 years in the Midwest [12].

### The socio-cultural impact of menstruation

Most people who menstruate experience menarche between the ages of 12 and 13 years [33]. Monthly menses recur for approximately 40 years, constituting over 500 menstrual cycles during an average lifetime [33]. While views about menstruation vary widely across cultures [34–41], health and social science experts worldwide recognize menstruation management as a global public health issue exaggerated by social stigma, misinformation, and structural barriers [33, 38, 42–45], the effects of which can exacerbate medical issues [46, 47]. These medical issues may lead to severe pains experienced during menstruation due to cystic fibroids to pre-menstrual depression disorders which impact menstruating people regularly. Skipping a cycle can indicate pregnancy, stress, or underlying disease [46], yet due to negative menstrual stigma, some people feel uncomfortable discussing menstruation with their healthcare provider [47] resulting in possible imperative health information being withheld.

Stigma revolving around menstruation derives from ‘religious, philosophical and cultural traditions’ that are often rooted in shame [48]. As a nation of multicultural identities, there is significant work that could address how the confluence of various religions, incomes, and cultural traditions impact menstruators, specifically students in the United States. A study in the United States found a significant correlation between a student’s absence in school due to menstruation and their ability to learn being negatively affected [49]. Isolation caused by social stigma around menstruation perpetuates the idea of the bodily process as a negative and shameful experience [43, 50].

In an updated definition of what constitutes menstrual health, Hennegan et al. [51] noted “Menstrual health is a state of complete physical, mental, and social well-being and not merely the absence of disease or infirmity, in relation to the menstrual cycle.” (p. 32). They additionally noted several factors pertinent to creating a social structure where the well-being of all menstruators is addressed. Among others, these factors include their ability to access accurate and timely understanding of their bodily functions around menstruations, and accessibility to safe, affordable, and appropriate products. While activists and scholars in several high-income and low-income countries have done tremendous amount of work to increase access and information for menstruators, they all agree that free menstrual products are not the only solution to create an equitable society for menstruators [10, 12, 52, 53]. Studies have shown that factors like access to bathrooms impact menstrual health management and must be equally addressed [9, 54]. In addition, a study conducted with women about their menarche reflected that lthe negative language and discourse around menstruation continues to impact how women manage their menstruation into adulthood [43]. These studies vouch for better information, knowledge, and the need to eradicate stigma and shame around conversations about menstruation.

Considering the significant socio-cultural impacts of menstruation and the effort to make accessibility of products as one of the many goals to bring equitability to HICs, this study attempts to highlight how experiences of menstruators are intertwined with the socio-cultural conversations around menstruations, such as accessibility of products in a university setting. Qualitative methods were used to provide an in-depth understanding of the impact of period policies and associated programs on product access including an analysis of personal, social, and cultural norms for those who menstruate.

## Methods

### Setting

Purdue University in West Lafayette, Indiana announced that pads, liners, and tampons will be provided in women’s and gender-neutral bathrooms of all administrative and academic campus buildings at no cost to users. This initiative was supported by at least three different [University] student groups, individual student petitions, and the faculty senate [55]. This announcement has been followed by a program in the city of West Lafayette to provide free menstrual products in buildings “throughout the city,” including parks and the West Lafayette School Corporation. These programs have been implemented to break down barriers that can impede the education and health of menstruaters [56].

### Participant recruitment

As part of a larger study, participants were recruited through direct email to university listservs and printed flyers placed in various university buildings. Recruitment began in October 2020 and continued through November 2020. All recruitment messages contained a link to an anonymous web-based screening survey to determine eligibility. Participants included currently enrolled students, who were assigned female at birth, were 18 years and older, and had experienced menstruation in the last six weeks. As participant recruitment happened when the SARS COV2 lockdowns shut down universities, recruitment majorly relied on direct mail from university listservs which hampered outreach to a larger population as could have been in an in-person recruitment drive.

### Virtual focus group discussions

Researchers conducted 5 90-min virtual focus group discussions with students enrolled at a Purdue University in the United States. Each focus group contained 5 – 8 participants; a total of 32 participants were enrolled in the study. All participants had experienced menstruation in the six weeks leading up to study enrolment. The mean age was 21 ± 2.38 years (range = 18 – 28). All participants self-identified as cisgender women, while 21 (65.6%) self-identified as White, and 6 (18.8%) as Asian, with the remainder self-identifying as other races, such as 1 (3.1%) Black. The average age number of cycles during the past 12 months was 11.87 ± 0.67 (range = 10–13). Participants were recruited through direct emails to university listservs and printed flyers placed in university buildings. Recruitment began in October 2020 and continued through November 2020.

Focus groups were captured using university recording devices, including a primary device and a secondary for backup. One member of the research team collated best practices for virtual focus group discussions and shared it with the team as part of the team. Two members of the research team facilitated each focus group. The first researcher acted as the primary moderator while the second acted as moderator, note-taker, and additional support. Prior to each focus group interview, researchers presented an informative overview of the study, answered questions, and obtained informed consent from the participants. Researchers initiated a discussion by asking probing questions about menstruation attitudes and behaviors and in-depth perspectives on the university-wide free menstruation management products, policies, and programs. Researchers asked questions relating to community attitudes, such as, “How do members of your community typically talk about menstruation?” and questions relating to policy impact, such as, “How does having access to free menstruation products on campus make you feel?” These interviews were semi-structured to allow for a free flow of conversation. In this way, researchers enabled participants to explore unique and shared ideas and experiences related to menstruation and the university-wide program. Participants received a $30 gift card for their time and efforts after the virtual group discussions. Purdue University’s Institutional Review Board (IRB-2020–811) reviewed and approved this study.

### Data analysis

Researchers utilized thematic analysis, a widely used, accessible, and flexible framework for systematically analysing qualitative data through identification, organization, and analysis [57, 58]. Researchers first conducted immersive, full content review to ensure familiarity with all data [57]. During this phase, researchers noted immediate patterns or ideas for potential codes and themes [57]. Following familiarization, researchers utilized a ‘*dualistic’* deductive/inductive approach for codebook development to allow greater representation of the data during the coding process [59, 60]. Initial codes were generated deductively and compiled into a preliminary codebook draft [60]. The inductive component permitted researchers to modify or add codes to better capture emerging themes from participant responses [60]. Coding was performed using HyperRESEARCH 4.5.1 [61]. Researchers completed multiple rounds of coding giving all data *‘full and equal attention*’ until saturation was reached [57].

Following the coding process, data were collated into potential themes and subthemes. Theme development was data-driven and closely reflected participant responses [62]. All co-authors reviewed resulting themes. Researchers thoroughly and collaboratively discussed and analysed individual themes and incorporated relevant subthemes to provide structure and differentiate levels of meaning [57]. Any discrepancies were resolved via consensus discussion and data review until final themes were fully agreed upon.

## Results

Overall, two sets of themes emerged from the data. In the first set, the participants discussed the value of free access to menstrual products in public spaces, and their (un) awareness of the Purdue University free period products policy. In the second set, the participants suggested ways to increase awareness of the free period products policy and the broader impacts of free tampon and pad policies and programs. Themes are presented below with illustrative quotes and noted by focus group number (FGX). A synopsis of the themes are captured in Table 1.

**Table 1:** Findings

***Overarching themes***	*Impressions of a Free Period Product Policy*	*Linking Free Period Products to Cultural Change*
*Subtheme 1*	The culture (and need) of product sharing during emergencies	Feeling supported by the university community
*Subtheme 2*	The need for appropriate products for comfort	Broader cultural impacts of the access to free products
*Subtheme 3*	The need for clear communication about the policy for wider access	

### Impressions of a free period product policy

#### The culture (and need) of product sharing during emergencies

The focus group discussions suggest that not having a menstrual product available when needed is a common experience, often due to the unpredictable nature of periods. One participant articulated it clearly,

*“I was at the mall in a bathroom and a girl in the stall next to me, I think she heard me opening a pad, and she asked me if I had an extra one because she didn’t have one, and started her period, I was kind of happy that I was on it cause I was able to help her out, you know, kind of not leave her hanging there. because I’ve been there.” (FG5)*

Settings such as malls, airports, classes, gyms, and national parks were all mentioned as locations where participants or people around them had been caught without a menstrual product and, as a result, had to rely on strangers to access it. Some participants expressed positive associations with helping one another out, and even saw it as a bonding experience. One participant shared,

*“Yeah I’ve been asked, by friends at school, but I’ve also had strangers, and in public every once in a while, which is also the reason that I almost always have a tampon on me like in whatever bag I’m carrying ((laughs)). Yeah, it feels pretty awesome to help someone when they are freaking out. (FG3)”*

This highlights positive aspects and the sense of community among menstruators. There were, however, negative feelings and anxiety surrounding product sharing as well. One participant shared,

*“I kind of have this weird uncomfortable experience with someone asking me for a product. Because I, since I’m always forgetting, I usually have a certain amount, and this one time someone asked for a product, and I was on my period. And I was like, okay, I only have one pad left so, do I give it to her? Or do I keep it so I’m able to use it later? But I ended up giving it to her, but then I had to like, go above and beyond to find one for myself later on.” (FG4)”*

This suggests that a lack of product access is not always an easy fix and can cause additional stress among menstruators. Another participant did not want to seem *“weird or… intrusive”* (FG1) while another participant shared,

*“I feel bad asking someone because I know that they are expensive. So, and I know that they paid for that. And because it’s not really free many places. So, like, I just feel bad, like asking for it. Like I’m sure many people would be like, be completely fine. If somebody asked me I would be fine. But so that, I don’t know. (FG2)”*

This highlights the additional barriers surrounding product access. Another participant shared, *“Having the ability to have coins also at the moment, can make it very complicated so if they are provided for us that would be good” (FG3).* This indicates that making something accessible goes beyond making it available: requiring coins or funds to use a dispenser can be a barrier to accessing products. Regarding access, one participant stated, *“If I will describe my feeling it will be frustration. Not just because I have to give mine. I don’t mind sharing that, because it should be like something that we could find anywhere but it’s not like that” (FG3).*

However, another participant shared why they would ask a stranger over buying poor-quality products from the dispenser machines in some bathrooms.

*I’ve always kind of gone above and beyond to make sure I was prepared so I wasn’t pushed into that corner where I had to rely on this machine. And I’d even go as far as to say, I’d rather ask a stranger for a product than pay for a machine just because I know that what I get from somebody else is probably going to be better than what I get from the machine. (FG5)*

Another participant stated*, “good quality products for free in one of these machines could help many students who are trying to make ends meet” (FG5)*, summarizing the overall feelings of the focus groups. There is a need for dispensers to provide product access, but they require conscientious and informed implementation to be effective in providing useful products for menstruators.

#### The need for appropriate products for comfort

Focus group discussion participants were also involved in product testing in this study. These participants highlighted the desire for higher quality products to be stocked in the dispensers on campus. High quality products were described as pads and tampons that are easy applicators and high absorbents. One participant shared,

“*I think that the quality of the products is important for people who, I don’t know, they depend on those products being accessible, all the time. And I just think that the quality becomes so much more important because, I feel like everyone deserves to be comfortable.” (FG5)*

Other participants echoed this sentiment, with one stating *“I guess having a known brand or at least one that is comfortable and know that even if it’s not well known. Everyone will feel good with it. I think it’s very important” (FG3).* One participant even stated, *“displaying the brand and product would make a big difference!” (FG5).* Along with quality concerns, there were environmental concerns expressed with one participant sharing, *“So just finding a way to create less waste with it is important to me”* (FG2) and following that up with, *“and especially if you are using less packaging, less waste, you can fit more product into a space than if you’re taking up that extra room with any unnecessary packaging as well” (FG2).* However, one participant saw this as a lesser priority, stating:

*“…in terms of emergencies, which is why those vending machines are there, I would think that environmental concerns would be the least thing you know, as the last priority one would have when they’re like, find out that oh, god, I’m on my period.” (FG1)*

The dispenser conditions were also discussed. Comments such as* “I think a while ago, when I saw those free products, they were just piling the exit of the vending machine?” (FG1)* and *“yeah so one time they didn’t have any” (FG3)* highlight the need for proper maintenance and restocking of dispensers for effective implementation. Decision-making was also brought up when discussing the quality of current products provided in the dispensers. One participant expressed their thoughts on the products, saying.

*“…but then, I don’t know I just feel like because it’s free whoever like makes these decisions to buy these products, they don’t really take and consider women’s health, they just find it. Like the lowest thing, cheapest thing they could buy and just stock it up there.” (FG5)*

These preferences for products led to the question, who should be making the product decisions for purchase? When asked, a participant shared, *“I think like anyone who has a period or has experienced it should be making those decisions because you can’t fully understand what it’s like or the situation unless you've kind of been through it” (FG2).* Other participants shared similar sentiments preferring that product procurement decision were made by individuals with direct menstrual experience and familiarity with the range of product options available. Participants gave various opinions regarding what products should be stocked in the dispensers. Generally, participants seemed to agree that *“[they] would prefer not to have cardboard applicators on tampons” (FG5).* For pads, one participant stated, *“the pads need to be thicker” (FG1).* However, this was met with some dissenting opinions, with another participant following up, *saying “actually, I have a contrary opinion. I think the pads are the thickest I’ve ever seen. It’s really diaper-looking. I think they should be thinner in my opinion” (FG1).* Contradictions like these highlight the need for product options within the dispensers. A participant capitalized on this, sharing, *“Not all of us prefer pads, not all of us prefer tampons. And, so, if we have the opportunity to have the option to go for the ones that we feel most comfortable with” (FG3).* Having quality choices within the dispensers can alleviate this fear of cheap or irritating materials that may not provide the desired absorbency, as this program aims to ease the stress and burdens menstruators face. When location was brought up in the focus group discussions, expansion into all restrooms was the main topic of discussion. One participant expressed,

*I do think it is important that like maybe it’s offered in, not just the women’s restroom. Just because there are people who don’t identify as women who do menstruate, and like, either having that in like a common area or just making it more accessible for everybody. (FG3)*

Another participant echoed this sentiment, stating, *“This is a little cost forward, but I would hope that they just put them in every bathroom, cause, you know, like, not just like some girls, it’s all of us” (FG4).* This push would help eliminate period poverty for everyone on Purdue University’s main campus by increasing accessibility to the program.

#### The need for clear communication about the policy for wider access

This study found that many students were unaware of the policy enacted in February 2020 approving Purdue University’s main campus to stock free menstruation management products in all women’s and gender-neutral restrooms in academic buildings. Some participants knew there were free products in dispensers around campus but were not aware of the policy. Participants reported accidentally finding the dispensers in a university building restroom and appreciated their presence but noted that they had missed the news around the policy. One participant shared *“I live in the dorms, and I haven’t seen any of the machines” (FG4),* highlighting confusion on where the free products can be accessed on campus, which did not include residence halls. Some participants were aware there were dispensers in academic buildings but weren’t aware of the policy or that the products inside were free. One participant shared, *“I honestly didn’t know those were free, I just kind of thought they were old machines, like still cost 25 cents” (FG2).* Another echoed this sentiment, suggesting *“people just assume they’re just old machines that never got removed.” (FG2).* These quotes indicated that while a policy was launched, there was need to increase awareness about the products within the university campus. Specifically, as Purdue University had changed the old machines to free product machines, students reported being unaware about the change due to lack of advertisement. One participants specifically suggested using social media as a tool to promote the change on campus,

*“Yeah, I agree, definitely putting stuff in the bathroom and maybe even, doing stuff online too. I follow a ton of Purdue University accounts that Purdue University runs for campus, so maybe having them post about something, or like sending out an email about it just so everybody knows because yeah I don’t, I don’t think I was aware of [the altered machines], either. (FG3)*

Increasing awareness of the period policy including when and where to access free products may lead to further positive experiences and feedback from menstruators on campus. When asking focus group participants how to raise awareness of this program and policy, one person stated,

*“Maybe a magnet near or around the trash can inside of the stalls would be nice. Since you might already be looking there if you forget that you have a product or if you forget a product.” (FG5)*

Others agreed with this form of advertising, with one participant emphasizing, *“The inside of bathroom stalls would attract some attention I think” (FG5).* Another participant echoed this, stating,

*“So definitely in the stalls if, because that’s really where the stress starts right when you’re in the stall. So, then you see the magnet, and you think, okay, I’m covered. I don’t have to leave. I’m still here. I’m going to be okay.” (FG5)*

Some focus group members expressed the desire for signs to be displayed in other areas of the women’s and gender-neutral restrooms to reach the attention of everyone within the bathroom, not just those utilizing the stalls. One participant shared, “*I think on the machines would be best” (FG5).* However, in a different focus group, a participant expressed that they did not think the old dispensers looked sanitary enough and commented, “Yeah, maybe they could do something about that, to at least make it look sanitary” (FG1).

Commenting on promoting the availability of free menstrual management products, other students added that there may be other places in the bathroom where the university can advertise other than the old dispensers. They added,

*“Yeah. Going off that I would say the best place to put the signs would be like between the mirrors where they have the abuse signs. But like, while you’re washing your hands, like even if you’ve read it a million times, you always just like reread it. So, if it says like, Purdue University’s going free tampon, then at least all the girls eventually will know cause everyone’s gonna wash their hands once.” (FG4)*

One participant emphasized the importance of signage size, stating that the current labels *“[are] so tiny to, know that it's free, like you would you have to actually walk up on it and see” (FG4).* Another participant mentioned an idea to use appropriate verbiage on these hypothetical advertisements, contributing, *“and just like having a note that these are for everyone, and take as you need them” (FG3).* Implementing wording like this can help ease the hesitancy some may experience when wanting to utilize a free menstruation product dispenser, as the intended use and target audience were shown to be unclear to some. This hesitancy was highlighted by one participant who stated*, “yeah, so my biggest concern also is the whole feeling guilty when I do have like my own access. And if I don't need it, I'm not gonna take it” (FG4).* Mass advertising outside of the restroom can also mitigate this uncertainty when dealing with the dispensers.

### Linking free period products to cultural change

#### Feeling supported by the university community

The feedback from the focus groups on Purdue University’s period policy was overwhelmingly positive. One participant mentioned feeling *“excited when Purdue University began providing free menstrual products in bathrooms” (FG4).* Another highlighted the novelty of a period policy, stating, *“I saw in the restrooms they have free tampons and pads if you need it. I’ve never seen that in another place. I thought that was really cool when I first got here”* (FG4). Another participant articulated that the free products provided mental security,

*“It provides that mental security that if you’re out on campus, and you’re surprised by your period, there is a place that you can go and you can get a product if you don’t have one and you don’t have to try to make do until you can get home or get to a store.” (FG2)*

Participants noted that not having access to period products on campus can make menstruators have uncomfortable situations, sometimes forcing them to choose between their education and going home to get a product. Other participants shared feelings of support and reduced stress. A consensus found in the focus group discussions was that providing free period products on campus made participants feel *“safe to know that [they] have an option to get some” (FG4) and “supported by the community and…it felt good to be reminded that this isn’t something that [they’re] totally on [their] own about” (FG1).* Another participant sharing echoed this, *“It’s just free for anybody to use and it’s super nice knowing that there’s so many people supporting you and you’re not being judged, to go grab one if you need anything” (FG2).* Providing free menstrual products in campus restrooms supports community menstruators and helps open conversations and decrease stigma surrounding menstrual health.

Some participants suggested that the free period product policy at Purdue University indicated that attitudes regarding menstruation are becoming more open. Participants specifically reported that before coming to the university, they experienced social stigma around menstruation. One participant shared that the *“connotation [of menstruation] changed once I got to Purdue University” in the way that the people here are “very open about it” (FG2).* This sentiment was shared repeatedly: *“at university, I think I’ve had more conversations about periods and menstrual products than I have before just because I’m in contact with people that are comfortable talking about it” (FG5).* A participant mentioned that a period policy is *“good for kind of normalizing it around campus”* (FG4). While the overall feeling was of Purdue University becoming a more period-positive space, one participant shared that the region where the university is located still bears some discomfort around the topic of menstruation,

*“but coming here to Purdue University and Indiana, there is definitely a different culture regarding menstruation, still not necessarily like stigma, but uncomfortableness with talking about menstruation, especially with, other men. In the male community, they just don’t want to talk about it at all. They don’t want to acknowledge it at all. So, it’s just harder to go about talking about it. (FG1)”*

This indicates that further work is needed to decrease stigma and expand open conversations regarding menstruation at this university.

#### Broader cultural impacts of the access to free products

The impact on access and culture at Purdue University suggests that broader impacts could be achieved as other universities and communities implement similar programs. One participant said, *“if they’re available and it’s more widely talked about, it’ll just be a better experience overall” (FG2).* Another participant phrased the conversations and studies around menstruation as progress,

*“There’s been progress. Studies like this happening to provide more and better products are available, and I think that’s something that’s definitely becoming more regular in a lot of places like schools and companies, just the access”* (FG3).

This highlights the broader cultural shifts as product access continues to be expanded and further normalized. Even so, stigma and shame surrounding menstruation run deep. One participant shared,

*“Even though a lot of people are like, yes I’m comfortable about it, I know I have maybe subconscious level of shame where it’s like, this isn’t something that everybody thinks is normal and I should conform to that idea” (FG3).*

Another participant rejected this perception and said, *“as you talk to more people it becomes less of a secret, I’m kind of wondering, what does it matter?*” (FG5). This highlights the importance of open dialogue around menstruation to further destigmatize it. Menstruation is normal and the focus groups recognized an overall shift in the attitudes toward it. One participant said, *“it’s definitely more open and the more popular female empowerment becomes, it’s just becoming so normalized now, which is a really nice thing” (FG2)*. As access and conversations expand, communities can make progress in normalizing and safely managing menstruation.

Many participants expressed gratitude for implementing the free menstruation management product policy and program on Purdue University’s campus. Participants expressed interest in having the general public implement similar policies to assist ending period poverty and making products accessible for everyone. One participant shared, *“…because in a really fantastic world or whatever I wouldn’t have to bring tampons and pads with me and a little bag everywhere I go and it there’s just in the bathroom”* (FG3). Implementing free menstruation management products in public restrooms is a great way to support menstruators in the community.

## Discussion

This study set out to explain how menstruator experiences are intertwined with the socio-cultural conversations around menstruation, such as accessibility of products in a university setting. Researchers conducted virtual focus group discussions to understand menarche and menstruation attitudes and experiences and the impact of a community-based period policy at a large, Midwestern university. Through the focus groups, we identified that students reacted positively about access to free menstrual management products and policy implementation and reported broader cultural impacts of access to free products in their university. Participants provided insights into how the current menstrual product program could be improved moving forward by suggesting promotion strategies and highlighting the implications of the policy at a community level.

This study emphasizes and supports the need to access menstrual products in public spaces. However, nearly half of the participants in the focus group discussions were unaware of the period policy launched in early February of 2020. As will be acknowledged in the limitations, due to SARS COV2 pandemic campus closures in March, students did not have enough time to explore the campus and know about the policy. However, the closures afforded researchers some time to conduct product testing of the menstrual products and identify unique ways to promote the policy as well as understand the socio-cultural impact of free menstrual product policies. As the policy was in its early stages, the champions for the policy saw as opportunity it receive feedback on the types of products they should place in the dispensers as quality of products continue to be an important cause in the goal to achieve period equity [63]. One of the major findings from product testing was the students’ preference for eco-friendly menstrual products which is in line with the findings recent studies which project that the younger generation is interested in eco-friendly products [64, 65].

These conversations on quality led to further feedback on the larger infrastructure around menstruation management. First, participants expressed apprehension about using the product dispensers due to past experiences where they needed to pay for the product which sometimes was empty and did not dispense the products. Participants also emphasized the importance of spreading awareness of the modified dispensers and dispenser sanitation and upkeep. These comments align with findings from other studies that free products succeed when adequate infrastructural developments complement the change [20]. Participants who were aware of the policy or placement of free menstrual products offered positive feedback and mentioned feeling supported by their community. Second, the study’s findings suggest that advertising a period policy through social media, magnets, and flyers could effectively increase awareness and use of the policy. This finding aligns with other studies that advertisements for the policy contribute to observational learning and behavioural modelling, which promote the use of these free products [20]. Openly discussing menstruation management in public spaces could help reduce the stigma menstruators face [43, 46], improve self-efficacy, and thus allow for better product access.

These findings suggest great potential for period policies among other universities and in community settings, which can be guided by Purdue’s senate resolution noted above. Along with champions and financial support as highlighted by Gruer et al. [15], this study also highlights the importance of gaining buy-in from the larger community. Similar policies can be implemented in other communities to increase access and reduce stigma, specifically by including feedback from the community. While menstruation is often perceived as a shameful phenomenon [43, 46], this study suggests that period policies help to normalize menstruation and make it easier to talk about. These conversations are crucial because making cultural changes and/or addressing social needs begins with a conversation [7].

These goals are evident in ongoing advocacy for better menstrual health and hygiene, which demands that these topics are included in conversations about health on both local and global levels [11, 66]. Nearly all 50 states have seen legislation introduced to improve menstruation management safety and access [67]. There are also calls for global standardization of these policies [13, 30]. Scotland has led the charge of making all menstrual products free across the country “for those who need it” and a study reported an increased uptake by people needing menstrual products. They also highlighted that Scots preferred eco-friendly products and better advertisement of the scheme so that those who need the products an seek them out easily [10]. Open conversations help cultivate more positive menstrual experiences, a better understanding of menstruation, and increased advocacy for menstruation management needs.

### Implications

This study presents various key considerations for successfully implementing an effective free period product policy and program in a university setting. More evidence on university students’ experiences with menstruation and the value of free menstrual products can serve as a useful resource for policymakers trying to make state-level legislative change. While there are several studies and legislations specifically targeting middle and high school students, the findings from this study can provide an example for other universities seeking to replicate such efforts at new campuses. Participants asserted that it is important to have a menstruator or someone who has experienced menstruation in the position that makes decisions regarding policies and the products they supply. While it is reasonably impossible to provide a full set of product options due to budget constraints [15], decisions on the product type and quality must be formed from an intimate knowledge of menstruation management. Knowledge gaps were identified among participants regarding awareness of the policy itself along with confusion about details such as location, intended use, and available products.

Advertising with flyers or magnets inside bathroom stalls, on product dispensers, between bathroom mirrors, online, and in highly trafficked areas of the university were all suggestions by participants to better inform the community. It is also important that the intended use and target audience are made clear in these advertisements to ease any hesitancy of taking products, along with the type and brand of product. If universities have old coin dispensers in place, they may appear unreliable to users. Therefore, advertising that the products are free and stocked should be ensured by the universities. Participants stressed the importance of product quality, variety, and brand, so these characteristics must be thoughtfully considered for effective implementation.

### Strengths and limitations

Qualitative methods led to an in-depth analysis of attitudes towards menstruation and methods in which menstruation can be safely managed. Limitations of this study include menstrual management products being restricted to pads and tampons. Other menstrual products such as period panties and cups were briefly discussed in focus groups but not provided for free in public spaces. Findings may be limited to settings or communities such as that of a large Midwestern university where the study took place, especially given that this study solely included student-menstruators and not employee-menstruators. Additionally, while we preferred to use the all-inclusive term, menstruators, our participant base consists of cis-gender women menstruators only. Future studies will benefit by examining the impact of access of menstrual management products by students identifying as transgender and non-binary. Despite limitations, the study provides insight into the feasibility and effects of period policies in communities.

Another major limitation of this study is that Purdue University launched the policy at the cusp of the shut down due to the SARS COV2 global pandemic. As students started to leave the campus and residential halls during the pandemic, the participants in this study could have experienced recall bias in remembering where and when free products were located. While the study was swiftly moved online to discuss product testing, promotional strategies, and the cultural implication of the policy, future longitudinal studies that track the policy’s implementation and impact will provide important insights for other universities, states, and countries looking at implying this policy.

Future research should investigate access to menstrual products in public spaces in other regions and communities and among diverse community members. In this study, we did not collect the participants’ socio-economic status. Previous studies conducted in public schools have highlighted that students from the minority communities experience higher level of period poverty and benefit from access to free menstrual products [10]. Further evaluation on how students from lower socio-economic backgrounds benefit from free menstrual product policy will expand our understanding. Additionally, as, participants in focus groups shared dissenting opinions on product preferences and quality, future research should also investigate which products should be provided to menstruators for an optimal experience. Further, future research should explore product dispenser sanitation and upkeep.

## Conclusion

This study offers insights into the struggles of managing menstruation in public places and the impacts of a program offering free menstruation products at a large, Midwestern university. Findings contribute practical recommendations to improve awareness, understanding, and implementation of a free product policy. The results suggest broader implications of period policies increasing access to products and supporting menstruators in universities and other communities by alleviating social stigma and positively affecting menstrual experiences.

## Data Availability

The datasets used and/or analysed during the current study available from the corresponding author on reasonable request.
